# The Experience of Deciding to Move to a Retirement Community: A UK Study

**DOI:** 10.1177/07334648251352591

**Published:** 2025-06-24

**Authors:** Kimberley J. Smith, Rachel Lawrence, Julie Round, Alison Benzimra, Andrew King

**Affiliations:** 1 School of Psychology, 3660 University of Surrey, Guildford, UK; 2 United St Saviour’s Charity, London, UK; 3 School of Sociology, 3660 University of Surrey, Guildford, UK

**Keywords:** retirement community, transition, decision making, moving, older adults, aging, ageism

## Abstract

Twenty-five older adults living in three retirement communities that catered to people from different sociodemographic backgrounds completed a semi-structured interview to share their experiences of making the decision to move to a retirement community in the UK. Data were analyzed with framework analysis, and three themes were generated. The overarching theme of maintaining independence and autonomy captured the importance that independence had in decision making. Theme 1 described the contextual factors that influenced older people when making their decision to move to a retirement community. Theme 2 encapsulated the importance of first impressions with a subtheme of existential and identity challenges capturing the tension that some people felt between wanting to move to a retirement community with the reality that this was a space for older people. The findings from this study are useful in highlighting the challenging circumstances that can influence the decision to move to a retirement community.

**What this paper adds**

- Gives a UK perspective to understanding the experience of deciding to move to a retirement community.
- Highlights the central role that maintaining independence and autonomy have in influencing the decision to move to a retirement community
- It is one of the first studies that shows how internalized ageism and identity challenges (in terms of identifying themselves as an older adult) can create a tension for older people when considering making a move to a retirement community.

**Application of study findings**

- Provides evidence that a person-centered approach to supporting older people with their decision to move to a retirement community could be beneficial.
- Suggests that housing providers should be mindful of not promoting independent living in a way that exacerbates existing negative discourse around older age and functional impairments.

## Introduction

Housing is a core component of age-friendly cities and communities, and to meet the needs of an aging population it is suggested that housing needs to be accessible, affordable, and safe (Nations, 2024; WHO, 2007). Retirement communities are one example of housing with support that older adults can consider a move to when staying in their current accommodation no longer seems feasible. These communities offer a specialized living environment designed to support older adults to maintain independence while also offering additional support and facilities (Gardner et al., 2005; Holland et al., 2017).

There is evidence that the decision to move at an older age can be different to moves at younger ages due to the life circumstances and support needs of older people (Sergeant & Ekerdt, 2008). Previous quantitative research highlights a wide range of contextual factors that can influence a decision to move to housing with support which include psychological, social, economic, health, and environmental factors (Franco et al., 2021; Roy et al., 2018). For people making the decision about moving to a retirement community, the most salient factors influencing a decision are often health-related with additional factors including bereavements, maintaining independence, loneliness, issues with home maintenance, and avoiding being a burden on family (Crisp et al., 2013; Gardner, 1994; Gilbert et al., 2015; Jungers, 2010; Walker & McNamara, 2013). Older adults can be attracted to retirement community living as it can be felt to offer features that they find lacking in their previous housing such as accessibility, safety, support, and a sense of community (Franco et al., 2021).

Different theories help develop our understanding of why an older person may make the decision to move. Developmental theories propose that the reason for moving in older age is linked to actual or anticipated major life events or transitions such as retirement, worsening health, and bereavements (Litwak & Longino, 1987). Person-environment fit theories propose that a move is precipitated by a person evaluating their needs and the support offered within their current environment, when the support offered within the environment no longer meets their needs then they can look towards alternative accommodation (Lawton & Nahemow, 1973). Another often-cited theory (push-pull theory) suggests that a relocation decision is not influenced by any single factor and instead is precipitated both by “push” factors (reasons that a person in pushed away from their current home) alongside “pull” factors (reasons that a person is pulled towards alternative accommodation) (Lee, 1966).

There is an interest in better understanding the decision-making process behind the move to a retirement community to better support people with this major life decision. However, most qualitative research that has examined the decision to move focuses on the North American and Australasian contexts, and there is limited evidence from the diverse types of retirement communities that we have in the UK (Carr & Fang, 2022). As such, the aim of this study was to explore the experience of making the decision to move to a retirement community in the UK.

## Materials and Methods

### Study Design

This qualitative study explored residents’ lived experiences of transitioning to a retirement community through semi-structured interviews conducted with residents living in three retirement communities across the south east of England. The communities were chosen to represent a range of socio-economic provision (see Table 1). This included means-tested social housing (social enterprise, charity, and local authority), private ownership (where a person buys their own housing), and hybrid approaches (composed of a community where a person could receive social housing, rent, or buy).

**Table 1.** Retirement Community Sites Included in Study.

Retirement community	Size	Types of housing on site	Facilities
Bridge Village	Approx 100 properties	Private ownership Independent living houses and apartments	Restaurant Gym Hairdresser Owners lounge Library Wellness center and spa Activity room Allotment and shared gardens Green space Guest suites Activities and classes Care and support services
Elmbridge village	Approx 250 properties	Hybrid housing Independent living houses and apartments for rent, sale, and social housing	Green space Wellness center and spa Swimming pool Hairdresser and salon Café Village shop On-site GP surgery Library Activities and classes Care and support services
The Grove	Approx 250 properties	Social housing Independent living houses Extra care apartments	Extensive green space Historic buildings Clubs and Societies Village shop Second-hand shop Post office Church Library Laundry area Activity center Therapy pool Bowling green Mini golf Village hall Club house Allotment and shared gardens Care and support services

### Participants and Recruitment

Eligible participants met the following criteria: aged 65+, lived in their community for a minimum of 12 months (to be able to reflect on the moving experience), and were able to participate in an interview. People with a diagnosis of dementia were excluded. A total of 25 residents were recruited, who had lived in a retirement community between 12 months and 20+ years (see Table 2). Participants were recruited using a convenience approach determined by the community (e.g., newsletter) which was facilitated by snowball sampling as recruitment progressed.

**Table 2.** Participant Characteristics.

Pseudonym	Time at village	Gender	Age	Marital status	Owners/tenants
Oscar	3–5 years	Male	70–79	Married	Owner
Beth	3–5 years	Female	70–79	Widowed	Owner
Evelyn	3–5 years	Female	80–89	Widowed	Owner
Isobel	3–5 years	Female	70–79	Widowed	Owner
Arthur	12–36 months	Male	80–89	Widowed	Owner
Edith	11–20 years	Female	70–79	Widowed	Owner
Peter	6–10 years	Male	80–89	Married	Tenant
Majorie	12–36 months	Female	70–79	Divorced	Tenant
Sylvie	11–20 years	Female	80–89	Widowed	Tenant
Kate	12–36 months	Female	70–79	Widowed	Owner
Ralph	12–36 months	Male	80–89	Widowed	Owner
Joy	3–5 years	Female	70–79	Widowed	Tenant
Elsie	6–10 years	Female	70–79	Divorced	Tenant
Ivy	12–36 months	Female	60–69	Divorced	Tenant
Joanne	12–36 months	Female	70–79	Married	Tenant
Marshall	12–36 months	Male	tbc	Married	Tenant
John	12–36 months	Male	tbc	Married	Tenant
Rosie	3–5 years	Female	60–69	Partnered	Tenant
Christian	20+ years	Male	80–89	Widowed	Tenant
Mary	3–5 years	Female	70–79	Widowed	Tenant
Agatha	3–5 years	Female	60–69	Divorced	Tenant
Cora	6–10 years	Female	70–79	Married	Tenant
Alice	12–36 months	Female	70–79	Married	Tenant
Theodore	12–36 months	Male	70–79	Married	Tenant
Pearl	6–10 years	Female	70–79	Widowed	Tenant

### Ethics

This study received favorable ethical opinion by the University of Surrey Ethics Committee: 19-20 049 EGA. Participants were provided with an information sheet and had the opportunity to ask questions before providing their consent if they were interested in taking part. Researchers reminded participants of their right to withdraw at any stage, and the names of a trusted other were collected at the start of the interview should a person become distressed (so the trusted other could come and support the participant). Protocols for safeguarding participants and the interviewer were also developed.

### Data Collection and Analysis

Semi-structured interviews were conducted to explore residents’ experiences in-depth. To cover the transitional experience, questions firstly asked about participants life experiences prior to the move and why they moved. Questions then centered around their experiences of the initial moving process and how they adjusted to community life. The interviews were conducted by using a telephone or video call between 2020 and 2021. Framework analysis (Goldsmith, 2021) was used to code the data and develop themes supported by NVivo. The lead researcher (KS) systematically coded the data for the first few papers alongside a second researcher (JR). Both researchers met to discuss their codes and agree on an analytical framework which was used to code all data and generate initial themes/subthemes. This framework was refined by KS with input from the study team, which was used to generate the themes presented in this paper.

## Results

**Figure 1.**
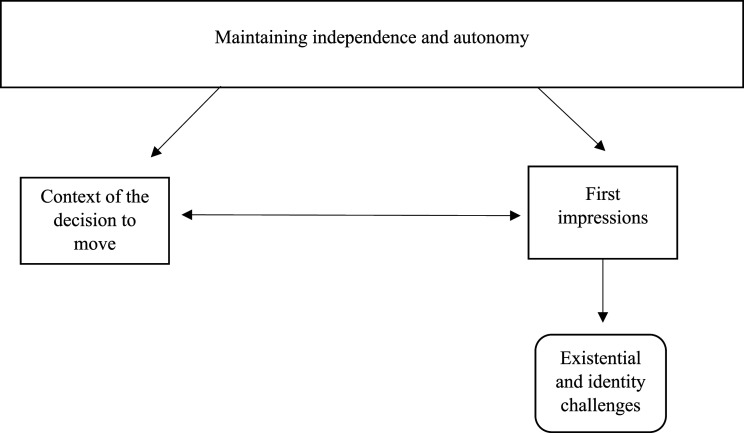
Thematic map. The thematic map demonstrates how maintaining independence and autonomy influenced both the context of the decision to move (by being a major reason that people were pulled towards retirement community living) and influencing first impressions (particularly how those who were less independent were viewed). The decision to move was influenced both by the first impressions that a person had alongside the context that their decision was taken in.

Three themes were generated (see Figure 1). The overarching theme of maintaining independence and autonomy captured the importance that independence had in decision making. Theme 1 described the multiple and overlapping contextual factors that influenced older people in making their decision to move to a retirement community. Theme 2 captured the importance of first impressions for older adults making their decision to move to a retirement community. The subtheme of Existential and Identity challenges outlined the tension created for some older people when faced with the reality of living in a community designed for older people. The thematic map outlines the linkages between these themes.

### Overarching Theme: Maintaining Independence and Autonomy

The importance of maintaining independence and autonomy was something that directly influenced older people in making their decision to come to a retirement community (theme 1) and their first impressions of the retirement community and older people (theme 2).

For many participants, retirement communities were seen as spaces where they could maintain their independence in an environment adapted for their current and future needs:

“That was it, that was it really because, er, from their prospectus, I know prospectuses are glossy sales leaflets, erm, I liked the idea of being independent. One day I will be ill. One day I will die but I’m going to put up a jolly good fight until then and I do not want anybody fussing over me, worrying about me unnecessarily and that’s what the [the village] mission statement, those dreadful things, said you know that I could be independent and I thought okay fine” (Beth, owner, Bridge Village).

Like Beth, many participants were attracted to the idea of retirement communities because of their focus on independence. Wanting to retain independence directly linked to participants wanting to make their decision to move autonomously, rather than feeling a decision had been forced on them (see Table 3, quote 1).

**Table 3.** Participant Quotes.

Quote number	Theme	Quote
1	Maintaining independence and autonomy	*“And um, and I, I realised er, found out that a lot of my ladies were living in their homes, they got very, very elderly. Um, and they ended up more or less being told, “come on mum you know, you need to go somewhere, we’ll put you here”. And they sort of didn’t have any say in the matter, they didn’t make choices. And so, they, they were very upset about it all and so, it really made that decision for me. I thought Isobel, you’ve got to do this for yourself, you’ve got to think about yourself”* (Isobel, owner, Bridge Village)
2	Maintaining independence and autonomy	*“ the, the purpose of moving was prompted by really my wife’s mother’s difficulties, and the, the problem with her leaving her existing house and to get her to go you know she could’ve gone somewhere, moved to a nicer place with a bit of sort of care and what have you which she absolutely resisted, and, in the end, you know it was like an enforced move into you know a prison camp almost [laughs] and she hated it. She didn’t like it then she wasn’t very well anyway by that time so it, what our motivation having been through that”* (Oscar, owner, Bridge Village)
3	Maintaining independence and autonomy	*“Yes, some people come in here, not by choice and they need a lot more, you know, reassurance and err things you know. It’s different, I think, if you came [of freewill] then you’re just really relieved and happy to be here”* (Ivy, tenant, The Grove)
4	Maintaining independence and autonomy	*“I mean it’s very beautiful looking and well kept, but then I was looking, and I was sort of seeing old people. This is me being really…I sort of saw old people, somebody in a buggy, and I thought oh God, yeah, it’s an old person’s place”* (Alice, tenant, The Grove)
5	Context of the decision to move	*“But, I didn’t mind, because in my mind I was thinking, ‘what am I going to do when I finish work, I want to live somewhere nice, I want to have the company?’, because you know, being… I’m widowed, and living on my own… well, it’s been 20 years now”* (Joy, tenant, The Grove).
6	Context of the decision to move	*“We just felt that [my husband] wouldn’t have to struggle to climb stairs, he would be safe, I wouldn’t be worrying that he would fall downstairs, you know, that we were in a safe environment -and you see, you’ve got, if you can understand me, when you live in a village in [the countryside], you don’t necessarily have streetlights -and when you get older, you need that feeling of safety -and this is what a retirement village gives you. It gives you that feeling of safety and peace”* (Sylvie, tenant, Elmbridge Village)
7	Context of the decision to move	*“My wife died in [redacted]. And I stayed, em we were staying in a, a four bedroom detached house, eh and eh, and so I stayed there and carried on staying there. It was too big for me to really but I hadn’t at that stage thought about moving to a retirement home or anything. What, what forced that onto me was I em, I had problems with em vascular problems in my legs. And, and one of my, my left leg eh went bad. Yeah and eh eventually, em, they had to amputate it above the knee. Yeah, so I, I started obviously thinking then about moving because where my house would have had to been adapted and it wasn’t going to be a, an easy to adapt eh, to get in and out the house, that is”* (Ralph, owner, Elmbridge Village)
8	Context of the decision to move	*“When I can’t get my socks on and need help or eh my wife’s got a problem, then they’re you know you’ve got, you’ve got care, you’ve got an emergency thing at night, there’s people here at night so that ticks another one of the boxes and the reasons for moving here”* (Oscar, owner, Bridge Village)
9	Context of the decision to move	*“ and your families, it’s a huge impact. The number of people you talk to here, and say the same thing. ‘The best thing about coming here was that the kids don’t worry anymore’*” (Rosie, tenant, The Grove)
10	First impressions	*“And, when I first heard about The Grove, mm, it was through a girl that I worked with that had somebody she knew in the village. Erm, and I actually applied before I knew that I’d need somewhere. I was living in tied accommodation”* (Joy, tenant, The Grove)
11	First impressions	*11a “But when you come into The Grove, you got lovely open spaces, most of the flats and cottages are nice; some of them are very small. Erm, the lovely open space and everybody was about your age”* (Christian, tenant, The Grove). *11b “Well, for me it was the fact that there’s quite a lot of open space. It’s not built round a square sort of thing. And there’s a, it’s quite open and also there was the, a doctor’s surgery on site em. You had a bar and a restaurant. Then games rooms if you so wished. There was a village hall. There seemed to be plenty to do and there’s a little shop as well. And a hairdresser [laughter]. So most of it was there eh”* (Ralph, owner, Elmbridge Village).
12	First impressions	*12a “Well, it was. When we came in for the first interview, the wife looked at me and said, “I wanna live here.” [laughs]. We got a calming feeling as you came through the gate”* (John, tenant, The Grove) *12b “gut instinct. I knew it was right and so did [my husband]”* (Edith, owner, Elmbridge Village)
13	First impressions: *Subtheme: Existential and identity challenges*	*“Before I’d got offered a place, I hesitated because I thought, do I really want to live with people that maybe they’re not well, have got mobility problems but then I have them, when my back flares up, I-I can’t walk for a couple of days. So, then I thought, well am I different? I’m not differen.”* (Majorie, tenant, Elmbridge Village)
14	First impressions: *Subtheme: Existential and identity challenges*	*14a “And told us to put our name down, I just thought, “God, yes you’re moving here, and this is where you will die.” Um and I just thought how do I get my head around that?”* (Joanne, tenant, The Grove) *14b And, I thought… and at the time, we all thought that was just the most fantastic thing, to think that you can come here, and you’re safe until you die”* (Rosie, tenant, The Grove)

For many people, having a relocation decision forced upon oneself was synonymous with moving to residential care which was a salient concern across different participants. This was often linked in participants minds to people leaving it so late that they would be too unwell to move to a place where they could retain independence. As such, making the choice to move to a retirement community was framed as a preferable alternative to being forced into residential care (see Table 3, quote 2).

Wanting to maintain autonomy and independence was a reason to move for some older people, but alongside this older people wanted the autonomy to make their own decision. Where people felt the decision to move was their choice there was a lot more satisfaction, whereas when they felt that they lacked autonomy in the decision this could lead to dissatisfaction and discontent (see Table 3, quote 3).

These narratives around lack of choice in the move were particularly evident in those people moving to subsidized housing. There was often a lot less choice in where people could move in a subsidized setting and what accommodation they could choose. For one person, the lack of choice she had in the move (which had to be made for financial reasons) was a source of notable discontent which impacted on the first impressions she had of the retirement community:

“I didn’t know how desperate it was and I drove round and I saw the proximity of people, and how close they were, and how close the front doors were, and all that sort of thing and I think it was beautiful weather and I just saw people sitting sort of in a static position outside their front door, um sort of oozing out and I said, ‘My god this is my idea of sheer hell’”(Joanne, tenant, The Grove).

In her interview, Joanne spoke of how she would never have chosen to live in a setting such as The Grove. Other people also struggled with seeing what they perceived as a lack of independence in people who lived in the retirement community when they made their initial visits (see Table 3, quote 4).

For many of these participants, seeing mobility aids was a sign of decline and dependence rather than tools that enabled older people to maintain their independence. The reality of these spaces being for older people who were perceived as being dependent conflicted with the idea of retirement communities as places that maintained independence.

### Theme 1. Context of the Decision to Move

Participants described different motivations, life events, and contextual factors that influenced their decision to move to a retirement community. Narratives highlighted that this had not been a simple decision, and older people were balancing the feasibility of staying in their previous accommodation with the potential benefits that could be offered by moving somewhere different. This balancing act would often be in favor of staying in previous accommodation until something happened where this was no longer seen as sustainable.

Participants commonly mentioned major life events that triggered their move such as a bereavement, health crisis, or relationship breakdown. Rather than the major life event itself directly precipitating the move, this event often heightened the resident’s awareness of broader issues or difficulties they were having in their current living situation. This was demonstrated by Beth (owner, Bridge Village) when talking about her husband’s death and the hedge:

“Erm, and so then he died and I was left in this beautiful Victorian house, lovely house, family house which required love and laughter of children. It wasn’t the sort of place for one person to live in so I had a look around. The thing that really made me decide to move funnily enough was the hedge….We had a big garden and he’d always cut the hedge. That’s what husbands are for and er, then when he died I had to get somebody else in to cut the hedge and they charged me £1,000.”

The cost and difficulties with home maintenance, particularly tasks that were her husbands’ tasks, together with additional narrative that she provided around not wanting to be a burden to her children made Beth re-evaluate her current living situation. Alongside highlighting potential difficulties with home maintenance as a lone householder, bereavements alongside other contextual factors such as retirement could also lead to people to become more aware of feelings of aloneness (see Table 3, quote 5).

These worries about being alone and feeling lonely led people to feel attracted to living somewhere set up to facilitate connections and companionship. Interestingly, a more salient draw to retirement communities across many of our participants was the sense of security and safety that these communities could offer. The type of safety and security that older people were looking for was often linked to difficulties that they had been experiencing in their previous accommodation such as financial issues, insecure housing, or health concerns. This is demonstrated by Ivy (tenant, Grove Village) who moved into subsidized housing after years of insecure housing:

“I just wanted to settle you know and as I say, I came and had a look and immediately the cottage option – the one downstairs from me was empty and the one I’m in now and the minute I walked in here, I knew this would be the better option for me…. I just didn’t want to be in a place where I was going to be unhappy and then have to re-move myself and find other places you know, I wanted to feel that I’d moved into somewhere where there was that security and obviously location was key and just to have the community erm and where there’d be company and you could make choices about where you wanted to fit on you know so obviously to contribute to the village, you know, that was what I was looking at doing, so it worked out amazingly for me.”

Ivys relief at finding a secure and safe long-term home in a community was echoed across different participants and was often linked to an awareness of the potential vulnerability that older age was perceived to bring. Participants felt that being in an environment adapted for their needs could help them feel less vulnerable while maintaining their independence. This was particularly evident in narratives around poorer health, which was the most salient contextual factor that influenced the decision to move amongst our participants (see Table 3, quote 6).

Alongside safety, most participants reported being drawn to retirement communities as they contained features (e.g., accessibility and on-site care) that were seen as useful for any health-related issues. When older people spoke of health and their move this could be a health crisis they (or their partner) had experienced, an awareness of declining health or an anticipation of potential future health decline. Some participants spoke about how they only made the decision to move when they were no longer able to make adaptations in their previous accommodation:

“well, we did try to move once or twice but it never worked out, and then we just stayed put and made alterations to the house and, but it was always, it’s a house with lots of steps in. But, erm, my husband, when he got Parkinson’s, gradually his legs got worse and worse. So, we got to the stage we thought well, maybe we’re going to have to move”(Kate, owner, Elmbridge Village).

Kate describes the balancing act that has been taking place between staying in her previous accommodation with meeting the needs of her husband. When the previous accommodation was no longer able to meet his needs, the balance tipped away from staying at home towards accessible housing. Many participants also described how health issues could be the major contextual factor that tipped the balance away from their previous home and towards a retirement community (see Table 3, quote 7).

Other participants described how moving to a retirement community before any major health events was seen as a way of being prepared for the future (see Table 3, quote 8). This awareness of needing to prepare for the impact of any future difficulties was also reflected in participants sharing that a further contextual factor influencing their decision to move was to avoid being a burden to their children alongside reassuring their children that they were safe (see Table 3, quote 9).

These narratives emphasize the multiple overlapping contextual factors that influence people in making their decision to move to a retirement community.

### Theme 2: First Impressions

A key part of making the decision to move to a retirement community was the first impressions that people had of retirement community living. For most people who owned their houses in a retirement community, their first impression of this type of living was formed through advertising materials:

“And as the years went by um, one day er, er, through the door came a card and the card er, had Bridge Village um, written on it. And the, and the er, picture of a swimming pool and I said to her, “that’s it, I’m going there when I get old” [laughing]” (Isobel, owner, Bridge Village).

Isobel’s first impression was positively influenced by advertising materials, and she was impressed by the lifestyle on offer in the village. On the other hand, those people who lived in subsidized housing (particularly those who applied for their housing through a charity or social care) were made aware of this type of housing through word of mouth or advice from social care professionals (see Table 3, quote 10).

Amongst people who lived in this subsidized accommodation, their first impressions were more around the practicalities of having somewhere to live rather than lifestyle. This could mean that the first impressions were often quite neutral and for these people visiting the community in person was often when their emotional first impression was created. For all participants, visiting their community was formative time in helping them make a decision. The physical elements of the community that people were particularly drawn to included natural and green spaces, the accommodation, and broader facilities. For facilities, it was important that these matched up to their health-related, safety, and social needs (see Table 3, quotes 11a and 11b).

Alongside the importance of these physical elements of the retirement community and their practical importance in forming a first impression, many participants also spoke about the feeling that being in the retirement community created for them. For many people, a sense of coming home or that the retirement community just felt right to them was a formative moment in their decision-making process (see Table 3, quotes 12a and 12b).

Interestingly, many of our participants from Bridge Village did not have the opportunity to visit their community as it was still being built and so their first impressions were based on advertising materials, building plans, and seeing the space where the community would be located. However, for those people who could visit their retirement community many of them spoke about how seeing a community full of older people challenged them emotionally. This is unpicked in the subsequent sub-theme.

### Subtheme: Existential and Identity Challenges

Many participants (particularly in The Grove) shared the impact of the first impression that they could be moving to a place that was for older people. Seeing older people in the retirement community often created a tension between the positive features that pulled them to this type of living with the reality of being with older people and being an older person themselves:

“ I just couldn’t see myself living there, ‘cause it was - I don’t know, it was, it was, um, The Prisoner, Stepford Wives and Waiting for God, Waiting for God all rolled into one, I had in my mind, ‘cause there’s all these smiley people, there was little go-, er, golf carts running around. [Laughs] I just thought - and on the gate as you go in, it said “for the elderly.” Well, I know I'm getting on, but that’s the last thing I wanted to think of myself as elderly... I felt like I was mixing with 100-year-olds when I’m only [in 60s], which really, in the real world is only 39 isn’t it” (Agatha, tenant, The Grove).

Agatha and other participants often spoke to an initial othering of people that were classified as “elderly” or more dependent. For many of our participants, it was seeing people using mobility aids that caused them to feel less comfortable and question whether they wanted to live in a place for old people. As mentioned by Agatha, people often had to challenge their own internalized ageism and their age identity when deciding whether they would like to move to a retirement community (see Table 3, quote 13).

The reality of being in a place for older people also caused existential challenges for some participants. This was a place that people would be living until they died, which could be a source of distress for some but comfort for others (see Table 3, quotes 14a and 14b). These narratives demonstrate how visiting the retirement community setting made people aware of their own mortality and identity as an older person.

## Discussion

This study is one of the first from the UK to examine the experience of making the decision to move to a retirement community. Our findings add to the evidence base by demonstrating the centrality that maintaining independence and autonomy have in influencing the decision to move to a retirement community while also demonstrating the complex contexts that influence a decision to move and how first impressions influence decision making. This study also makes an important contribution to knowledge by allowing us to demonstrate the internal conflict and tension created by the reality of deciding to move to a place for older people with their own age identity and the marketing of retirement communities as spaces to maintain independence and stay young.

Retirement communities often market themselves as spaces that allow older people to maintain their independence through active living. Therefore, it is not surprising that maintaining independence and autonomy were core factors influencing the decision to move to a retirement community. This finding has been echoed across qualitative studies in other countries where independence was an important factor pulling people towards retirement community living (Ball et al., 2009; Berglund-Snodgrass & Nord, 2019; Carr & Fang, 2022; Henderson, 2011; Kraft, 2002; Lo, 2000; Omoto & Aldrich, 2006; Walker & McNamara, 2013; Williams & Guendouzi, 2000). For instance, work from Carr and Fang (2022) generated themes of “prolonging midlife” and “future-proofing” that captured this idea that people were attracted to retirement communities as they could help them remain young and independent for as long as possible while also providing them with reassurance that they will be prepared to remain as independent as possible in the future by living in accessible accommodation with additional support. Being attracted to retirement communities as places could help one to prolong their midlife would also explain some of the negative reactions that our participants had to seeing people who were perceived as “elderly” (a term our participants sometimes used to other those they perceived as more frail or dependent). In addition, our work indicated that the most salient feature of autonomous decision making was that the older adult felt they had made a decision for themselves before it was forced onto them. This is a common finding across different qualitative studies (Ball et al., 2009; Chen et al., 2008; Harhaj, 2014; Walker & McNamara, 2013) and for many people is linked to a negative stereotype of people being forced into residential accommodation. Finally, wanting to retain their own independence was important in influencing the ways that viewing dependent older people impacted some of our participants. This was particularly notable in narratives where people othered people they perceived as being more dependent. We observed this finding more amongst people who moved to subsidized housing, but many of the owners we interviewed bought properties on sites that were not yet constructed and so would not have seen older people on site when they made their initial visit before buying.

We found there were complex contexts that underpinned a decision to move and that these were highly individualized. There were factors that acted to push people away from previous accommodation and pull people towards retirement community in line with push-pull theory (Lee, 1966). The most salient factor that pushed people away from previous accommodation was health, a finding in line with other research (Franco et al., 2021). In our study health was often the contextual factor that forced a decision to move, often because previous accommodation was not suitable or safe for their health and support needs. Whereas some of the other contextual factors influencing a decision to move (e.g., being a burden to children and loneliness) did not mean a housing environment became unsafe or unusable. Some of the most salient pull factors towards a retirement community echoed previous research and included safety, security, accessibility, and support (Franco et al., 2021). However, push-pull theory could be critiqued as simplifying the complex reasons that older people decide to move and not considering how different factors and life circumstances can compound one another in influencing a decision. While push-pull theory can be useful in helping to categorize some of the factors that influence a decision to move, it does not account for ongoing evaluation that our participants were undertaking in assessing their needs and accommodation. Instead, environment-fit models (Lawton & Nahemow, 1973) offered more of an insight into the balancing act that we saw amongst our participants in deciding when to move.

We also found that the first impressions created by the retirement community were fundamentally important in influencing a decision to move. While the initial “remote” impression of the retirement community differed between people who bought housing versus those who moved into subsidized housing, both groups shared the importance of visiting the community and the first impression that somewhere felt right. However, first impressions were complicated for some people by the tension created by existential and identity challenges. Previous qualitative work has highlighted that older people can speak negatively about visiting retirement communities due to their own associations of these spaces with death and decline (Green & Ayalon, 2019). For some people, it is the rhetoric that contradicts ideas of decline and aging (i.e., retirement communities as places where one maintains independence and stays young) that attracts them to retirement community living (Carr & Fang, 2022; Williams & Guendouzi, 2000). This could mean that residents experience the disconnect of expecting to live in a space that will keep them young and independent with the reality that this is a space for older people. Struggling with seeing a space full of older people was also linked to a person’s own age identity and othering those perceived as “older.” Existing research highlights that many older people feel subjectively younger than their chronological age (Barak, 2009) and that for many older people “older” age is demarcated by poorer health and functional limitations (Bowling et al., 2005). This could explain why older people who were perceived to be less independent due to use of mobility aids were othered as being older by those who felt subjectively younger.

### Implications

Our findings have important implications for housing providers by emphasizing the importance of independence to older people moving to this type of housing. However, by promoting their provision as places where people can stay young, this could inadvertently be feeding into a societal narrative that aging is a bad thing. This could also explain some of the psychological resistance experienced by some older people when seeing those they perceived as more dependent or frail. Instead, retirement communities could investigate ways of promoting their accommodation in a way that embraces the differences in peoples aging experiences and support needs. Our findings also demonstrate that the decision to move is often taken in a broader context that many people would find difficult (e.g., health difficulties and bereavements). As such, making housing providers aware of these complexities could mean they provide additional support to people with their decision.

### Strengths and Limitations

A strength of this study is the use of a qualitative methodology to explore the transition in significant depth. Participants were also recruited from different types of retirement communities which allowed us to unpick differences in the experiences of those people who buy their own house and those who are tenants in subsidized housing. The limitations include the study was restricted to the south of England and participants were mostly highly engaged in their retirement communities which would have influenced the themes generated.

### Conclusions

This study demonstrates that independence and autonomy are important drivers of the decision to move to a retirement community. Furthermore, our findings emphasize that a decision to move is influenced by complex and unique circumstances for every older person which are also compounded by the financial situation of the older person and the type of retirement community they are moving to. Finally, some older people struggled with the reality of retirement communities as spaces for older people with their own age identity as a younger person. Our findings have implications for the way that housing providers think about supporting older people considering making this move.

## Data Availability

Please contact the corresponding author regarding access to the data collected and analyzed in this study.*
